# The snoRNA target of t(4;14) in multiple myeloma regulates ribosome biogenesis

**DOI:** 10.1096/fba.2018-00075

**Published:** 2019-05-22

**Authors:** Vanessa Oliveira, Nitin Mahajan, Melissa L. Bates, Chakrapani Tripathi, Kyusik Q. Kim, Hani S. Zaher, Leonard B. Maggi Jr, Michael H. Tomasson

**Affiliations:** ^1^ Division of Hematology, Oncology and Bone Marrow Transplantation, Department of Internal Medicine University of Iowa Iowa City Iowa; ^2^ Division of Oncology, Department of Medicine Siteman Cancer Center, Washington University School of Medicine St. Louis Missouri; ^3^ Department of Health and Human Physiology University of Iowa Iowa City Iowa; ^4^ Stead Family Department of Pediatrics University of Iowa Iowa City Iowa; ^5^ Holden Comprehensive Cancer Center University of Iowa Iowa City Iowa; ^6^ Department of Biology Washington University St. Louis Missouri

**Keywords:** chromosomal translocation, hematological malignancy, protein synthesis, reactive oxygen species

## Abstract

The orphan small nucleolar RNA (snoRNA) ACA11 is overexpressed as a result of the t(4;14) chromosomal translocation in multiple myeloma (MM), increases reactive oxygen species, and drives cell proliferation. Like other snoRNAs, ACA11 is predominantly localized to a sub‐nuclear organelle, the nucleolus. We hypothesized that increased ACA11 expression would increase ribosome biogenesis and protein synthesis. We found that ACA11 overexpression in MM cells increased nucleolar area and number as well as argyrophilic nuclear organizing regions (AgNORs). Supporting these data, samples from t(4;14)‐positive patients had higher AgNORs scores than t(4;14)‐negative samples. ACA11 also upregulated ribosome production, pre‐47S rRNA synthesis, and protein synthesis in a ROS‐dependent manner. Lastly, ACA11 overexpression enhanced the response to proteasome inhibitor in MM cells, while no effect was found in response to high doses of melphalan. Together, these data demonstrate that ACA11 stimulates ribosome biogenesis and influences responses to chemotherapy. ACA11 may be a useful target to individualize the treatment for t(4;14)‐positive myeloma patients.

**Abbreviations**
AgNORs: argyrophilic nuclear organizing regions
BrdU: bromo‐2'‐deoxyuridine
Btz: bortezomib
MM: multiple myeloma
NAC: N‐acetyl‐cysteine
ROS: reactive oxygen species
snoRNA: small nucleolar RNA

## 1 INTRODUCTION

Chromosomal aberrations are frequently used to stratify patients with multiple myeloma (MM), and the t(4;14) translocation and 17p13 deletion are consistently associated with poor survival.^1^, ^2^, ^3^ In cells, t(4;14) is transposed upstream of the Nuclear Receptor Binding SET Domain Protein 2 gene (NSD2, previously named WHSC1 and MMSET), leading to its ectopic overexpression.^4^ NSD2 overexpression is found in all t(4;14) positive MM patients, and NSD2 knockdown induces cell death in MM cells lines. However, NSD2 overexpression of the protein coding sequence is not sufficient to transform primary cells.^5^, ^6^, ^7^

ACA11, an orphan box H/ACA class small nucleolar RNA (snoRNA, intron 18‐19 of the NSD2 gene), is expressed co‐transcriptionally with the NSD2 gene and is also overexpressed in all t(4;14)‐positive myeloma patients.^7^ Our laboratory has studied the role of this non‐coding RNA in MM pathophysiology and found that ACA11 overexpression increases reactive oxygen species (ROS) production via inhibition of nuclear (erythroid‐derived 2)‐like 2 (NRF2) factor and drives cell proliferation of MM cell lines and primary B cells, but not transformation.^8^ High proliferation rates require increased protein synthesis, supported by upregulation of ribosome biogenesis pathways.^9^, ^10^

The biosynthesis of ribosomes is a complex process involving transcription of components, processing, quality control, assembly and covalent alterations of pre‐RNA, and export to the cytoplasm.^11^ Post‐transcriptional modifications (eg 2′‐O‐Ribose methylation and pseudouridylation) in ribosomal biogenesis are mainly regulated by two classes of snoRNAs, box C/D and box H/ACA, respectively, and have been implicated in cancer biology.^11^, ^12^, ^13^, ^14^, ^15^ Interestingly in MM cells, ACA11 seems to interact with a small nucleolar ribonucleoprotein complex.^7^ ACA11 physically interacts with nuclear proteins associated with RNA processing, including RNA splicing factors (SF3B1, SF3B2, and SFPQ), ATP‐dependent RNA helicase A (DHX9), RNA‐specific adenosine deaminase (ADAR), and eight members of the heterogeneous nuclear ribonucleoprotein family (HNRNP). Furthermore, ACA11 RNA was found in both DHX9 and IL enhancer‐binding factor 3 (ILF3), which was detected in DHX9, nucleolin (NCL), ADAR and HNRNPU immunoprecipitates.^7^

Based on the nucleolar localization of ACA11, we hypothesized that ACA11 overexpression increases ribosome biogenesis and protein synthesis. As ACA11 overexpression promotes ROS accumulation, we also hypothesized that increased ribosome biogenesis is ROS‐mediated and that antioxidant treatment would reduce the effects of ACA11 overexpression in MM cells.

Here, we show that ACA11 overexpression increases growth in MM cell lines and induces phenotypic changes in the nucleoli of MM cell lines and human MM samples. Furthermore, ACA11 overexpression increases ROS‐dependent ribosome biogenesis and protein synthesis. Lastly, we show that ACA11 overexpression is a determinant of the response to chemotherapy commonly used in MM. Together, these findings demonstrate that ACA11 controls ribosome biogenesis associated with the t(4;14)‐positive chromosomal translocation in MM and is an important mediator in the response to chemotherapy.

## 2 MATERIALS AND METHODS

### 2.1 Cell lines source, plasmid constructions, and virus production

Human myeloma cell lines MM1.S [t(4;14)‐negative] and H929 [t(4;14)‐positive], were cultured as described previously or as per ATCC guidelines. ACA11 was acutely overexpressed in MM1.S cells. The chemically modified antisense oligonucleotide 2 (αA2) was used for ACA11 knockdown in H929 cells as previously described.^7^, ^8^

### 2.2 Patient samples

CD138+ cells isolated from the bone marrow collected from t(4;14) positive and negative patients were obtained from the Tissue Procurement Core Facility and Tumor Bank of Washington University in St. Louis, Missouri, USA.

### 2.3 Cell sources, culture conditions, plasmid constructions, and virus production

Human myeloma cell lines MM1.S [t(4;14)‐negative] and H929 [t(4;14)‐positive], were cultured as described previously or as per ATCC guidelines.^7^, ^8^ Briefly, MM1.S cells were cultured (density lower than 0.5 × 10^6^ cells/mL) in RPMI 1640 media (Gibco, Invitrogen Life Technologies, USA) supplemented with Glutamine (Gibco, Invitrogen Life Technologies) plus fetal bovine serum (FBS 10%) (HyClone, USA) and penicillin‐streptomycin (1%) (Invitrogen Life Technologies, USA). H929 cells were cultured in Dulbecco's Modified Eagle Medium media supplemented with D‐Glucose (4.5g/L), L‐Glutamine, FBS (10%) (HyClone) and penicillin‐streptomycin (1%) (Invitrogen Life Technologies). All cultures were routinely screened for *Mycoplasma *species using PCR Mycoplasma Detection Kit (abm Inc, Richmond, BC, Canada) and found to be free of contamination. For ACA11 infection, MM1.S cells (1 × 10^6^ cells) were seeded in six‐well plates, and then immediately infected with lentivirus (12‐16 hours). ACA11 infection was confirmed using RT‐PCR as previously reported.^7^, ^8^ In H929 cells the chemically modified antisense oligonucleotide 2 (αA2) was used for ACA11 knockdown and antisense nucleotide (ASO) targeting GPF was used as control as previously described.^7^, ^8^ All plasmid constructs and the method of virus production used in these experiments were described previously.^7^, ^8^

### 2.4 Cell number counting assay

After acute infection, MM1.S cells (1 × 10^5^ cells) infected with empty vector (pLKO.1) or ACA11 were seeded in triplicate in 24‐well plates and the cell number was determined every 48 hours by counting live cells by Trypan Blue exclusion (Gibco, Invitrogen Life Technologies).^8^

### 2.5 Measurement of nucleoli number and area

Asynchronously growing MM1.S cells infected with empty vector (pLKO.1) and ACA11 were fixed with 2% glutaraldehyde in phosphate‐buffered saline for 10 minutes. Samples were further processed by the Washington University Department of Cell Biology's Electron Microscopy Core. Pictures of nuclei and nucleoli were taken at magnifications of 4000X and 8,000X, respectively. Seven fields/grids were scanned, the number of cells (range 60‐100) and nucleoli counted, and the total nucleolar area per nucleus was measured using Imagej software (Wayne Rasband, National Institutes of Health, USA, version 1.52a).

### 2.6 Quantification of nucleolar organizers regions by silver‐staining

Human cell lines MM1.S and H929, and CD138‐positve bone marrow cells collected from t(4;14) positive and negative MM patients (Tissue Procurement Core Facility and Tumor Bank of Washington University in St. Louis, Missouri, USA) were smeared and air dried on slides and assessed for argyrophilic nuclear organizing regions (AgNORs) according to a modified protocol.^16^ Cells were fixed in 2% glutaraldehyde flowed by post fixation in ethanol‐acetic acid solution (3:1), stained using a formic acid‐33.3% silver nitrate solution in 0.66% gelatin, and then mounted on slides using Vectashield (Vector Laboratories, Burlingame, USA). AgNOR numbers in at least 100 cells on each slide were blindly quantified at 100x magnification.

### 2.7 Polysome profiling

MM1.S and H929 (5 × 10^6^) cells were treated with cycloheximide (50 μg/mL) prior to lysis, and fractionation was carried out over a sucrose gradient (7%‐47%). Gradients were fractionated, and RNA absorbance monitored at 254 nm to detect ribosomal subunits as previously described.^9^

### 2.8 Cell Mass

Cells (3 × 10^6^) were washed with twice 1x PBS and lysed and sonicated in RIPA lysis buffer. Total cellular protein content of clarified lysates was measured using the DC Protein Assay Kit (Bio‐Rad Laboratories) as previously described.^17^

### 2.9 [^35^S] methionine incorporation assay

MM1.S and H929 (3‐5 × 10^5^) cells were plated in triplicate and then starved of methionine and cysteine for 30 minutes. Cells were pulsed with 14.3 μCi of [^35^S] methionine (PerkinElmer Inc, Downers Grove, IL) for 4 hours and then immediately washed twice with cold PBS and lysed with Triton X‐100 buffer (1%). Total protein was precipitated from the lysates with 10% trichloroacetic acid. Pellets were subjected to liquid scintillation counting to measure the incorporated counts per minute as previously described.^9^, ^17^, ^18^

### 2.10 Cell volume

Cellular volume was calculated based on the average diameter of the cells measured (20 000 cells/condition) using a Moxi Z mini automated cell counter and analyzed with MoxiChart software (version 3.2, ORFLO Technologies, USA) using cutoffs of 8 and 19 µm.

### 2.11 2′‐*O*‐Methylation‐seq library preparation

Briefly, 10 µg of total RNA was fragmented by incubation at 95°C for 20 minutes in fragmentation buffer (12 mmol/L Na_2_CO_3_, 44 mmol/L NaHCO_3_, 1 mmol/L ethylenediaminetetraacetic acid; pH 9.2). The fragmented RNA was then run on a 10% polyacrylamide urea gel and fragments from 100 to 200 nucleotides were excised, eluted, and ethanol precipitated before resuspension in water. Fragments were ligated to a short adenylated DNA oligonucleotide, 5′rAppCTGTAGGCACCATCAAT/3ddC/3′, at their 3′ end using truncated T4 RNA ligase 2 (NEB). Ligation products were gel purified and then reverse transcribed, using AMV reverse transcriptase (NEB) and a reverse primer complementary to the linker (RS‐1, complementary sequence in bold). cDNA was gel purified to remove unused primer and then circularized (CircLigase kit, Epicentre^TM^, Palo Alto, CA). Circularized cDNA was then subjected to PCR using a forward primer and reverse primers with unique barcodes complementary to the RT primer (complementary sequences to RS‐1 underlined and italicized for the forward and reverse primers, respectively). Samples were gel purified, using native PAGE to remove primers and then analyzed for length and purity using Agilent Bioanalyzer. Libraries were then pooled and subjected to Illumina™ single‐read HiSeq 2500 sequencing. Sequencing was performed by the Genome Technology Access Center (GTAC) at Washington University in St. Louis, St. Louis, Missouri, USA. (Linker and Primers are in Table S1).

### 2.12 Bioinformatic Processing of Reads

Raw data was checked for quality and length using FastQC (https://www.bioinformatics.babraham.ac.uk/projects/fastqc/). Next, reads were demultiplexed using Novobarcode (http://www.novocraft.com/). The reads were then trimmed using Cutadapt v 1.16^19^ to remove the 3′ adapter. Reads without 3′ adapter or shorter than 15 nucleotides were discarded. The trimmed reads were mapped to the 45S pre‐ribosomal RNA sequence of Homo sapiens (RNA45SN5; NR_046235) using Bowtie2 v 2.2.5^20^ with default settings. Only reads mapping uniquely and with no mismatches at the last three nucleotides of the 3′ end were used to calculate methylation scores. Annotated modification sites were taken from the Ribosomal Modification Maps Database.^21^ Coordinates were lifted over to NR_046235 using a custom bash script. Meth‐Score for annotated sites was calculated as previously described.^22^

### 2.13 47S pre‐rRNA qRTPCR

MM1.S cells overexpressing ACA11 or H929 cells transduced with ACA11 ASO2 knockdown were treated with N‐acetyl cysteine (NAC, 1.0 mmol/L), as previously described.^8^ Following RNA isolation (RNAesy,Qiagen, Germany) first‐strand cDNA was prepared (iScript Kit, Bio Rad Laboratories, USA) and 47S pre‐rRNA levels were measured by qPCR as previously described.^9^

### 2.14 rRNA export assay

5 × 10^6^ MM1.S cells overexpressing ACA11 or H929 cells transduced with ACA11 ASO2 knockdown cells were starved in methionine/cysteine‐free media for 30 minutes prior to labeling with ^3^H‐methyl‐methionine (Perkin Elmer, USA) for 30‐minutes. Following a 2‐hour chase in complete media with excess cold methionine, nuclear and cytoplasmic exacts were prepared as previously described.^9^ RNA was isolated using RNA‐solv (Omega BioTeck, Norcross, GA), separated on formaldehyde containing agarose gels, transferred to Hybond XL (GE Healthcare Life Sciences, Pittsburgh, PA), crosslinked using a Hoeffer UV Crosslinker (Hoefer, Inc, USA), and sprayed with EN3HANCE Spray (Perkin Elmer). The dried membrane was exposed to preflashed autoradiography film with an Intesifying screen at −80°C for 1‐2 weeks. Autoradiograms were scanned using an ImageScanner III apparatus (GE Healthcare Life Sciences) and densities were determined using ImageQuant V V.2005 software (GE Healthcare Life Sciecnes, Pittsburgh, PA). The data are a representative image of three independent experiments.

### 2.15 Dose response to chemotherapy agents

MM1.S cells (1 × 10^5^) infected with empty vector or ACA11 were treated with bortezomib (Btz) (in nmol/L: 0, 1.25, 2.5, 5) or melphalan hydrochloride (in µmol/L: 0, 1, 10, 20, 30) for 48 hours.^23^, ^24^, ^25^ Then, the following assays were performed: (a) cell number: evaluated by cell counting assay using Trypan Blue exclusion; and (b) cell proliferation: using a colorimetric bromo–deoxyrudine (BrdU) incorporation assay, per the manufacturer's instructions (Cell Signaling Technology, USA). An additional culture of ACA11‐ or vector infected of MM1.S cells (1 × 10^5^) were treated with NAC (1.0 mmol/L), btz (5.0 nmol/L) or NAC (1.0 mmol/L) (Sigma‐Aldrich, USA) plus btz (5.0 nmol/L). After 96‐hours cell numbers were assessed using Trypan Blue exclusion and the data were represented as the change in cell number (change in cell number = [cell number after 96 hours minus cell number at time zero]/4).

### 2.16 Statistical analysis

Results are displayed as mean ± SEM or as fold change ± SEM. Statistical analysis was performed using Graph Prism (V5, GraphPad Software, La Jolla, CA). Mean values were compared using unpaired Student's *t* test or ANOVA followed by Bonferroni post hoc. When expressed as fold change, data were analyzed with a one sample *t* test with the hypothetical mean set equal to the control (pLKO.1/green fluorescent protein controls) (hypothetical mean = 1). The dose–response to chemotherapy was evaluated with a general linear model, in which the difference in slope and intercept were evaluated. Statistical significance was set a priori at *P* < 0.05.

### 2.17 Data sharing statement

For original data, please contact michael-tomasson@uiowa.edu.

## 3 RESULTS

### 3.1 ACA11 overexpression determines nucleolar phenotype

**Figure 1:**
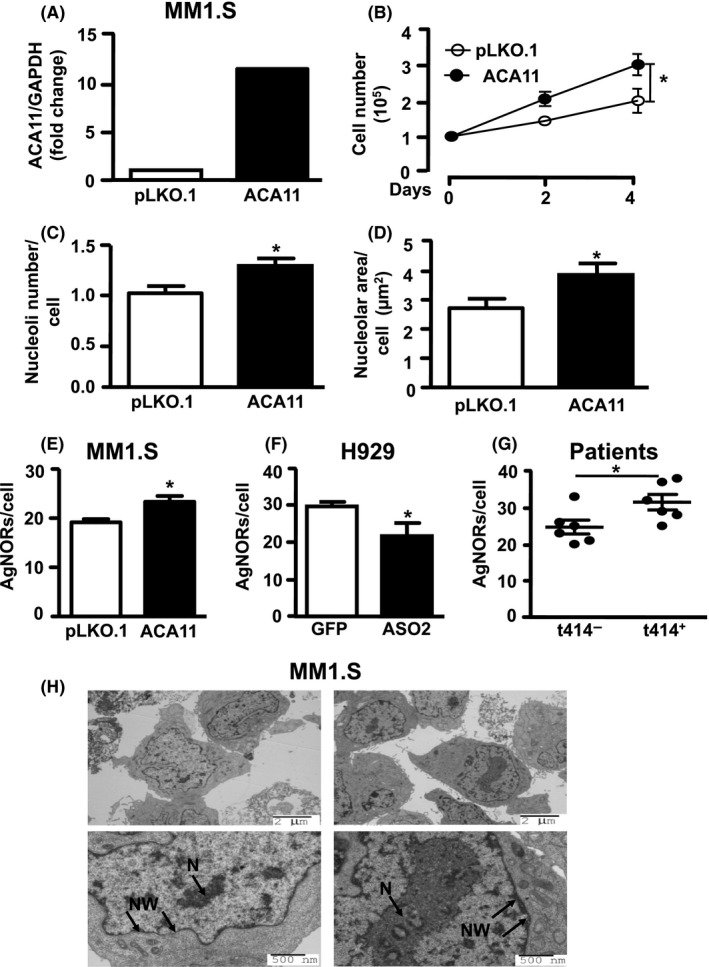
ACA11 overexpression alters nucleolar characteristics in multiple myeloma (MM) cell lines and patient samples. A, real‐time quantitative reverse transcription PCR analysis of ACA11 snoRNA expression levels in MM1.S cells relative to glyceraldehyde 3‐phosphate dehydrogenase. B, MM1.S overexpressing ACA11 exhibits cell number increase compared to MM1.S cells infected with empty vector (pLKO.1). C, Quantification of nucleolar number and (D) area in MM1.S cells overexpressing ACA11 compared to pLKO.1 control. E, Quantitative analysis of AgNOR (Argyrophilic NORs) number in MM1.S cells overexpressing ACA11 compared to pLKO.1 control (F) in H929 cells with ACA11 knockdown compared to green fluorescent protein control; and (G) in MM Patient samples with t(4;14)‐positive MM compared to t(4; 14)‐negative MM. *P < 0.05. H, Representative transmission electron micrograph from MM1.S cells used in the quantification in (C) and (D) showing the effects of ACA11 overexpression on nucleolar morphology. N, nucleoli, M, ribosomes at nuclear membrane. Top panels were imaged at 4000X magnification and bottom panels at 8000X

Corroborating previous data from our laboratory,^8^ acute overexpression of ACA11 in MM1.S t(4;14)‐negative cells increased ACA11 expression (Figure 1A) and increased cell number over 4 days in culture versus empty vector (Figure 1B, AUC: pLKO.1 = 5.89 ± 0.29 vs ACA11 = 7.98 ± 0.45, *P* = 0.03). Given that ACA11 is localized to nucleoli, we explored the potential effects of ACA11 overexpression in the nucleoli of MM cells. We found that ACA11 overexpression increased nucleoli number per cell (Figure 1C, *P* = 0.01) as well as nucleolar area (Figure 1D, *P* = 0.03). Nucleolar organizer regions (NORs) are chromosomal domains, which contain ribosomal DNA genes and are associated with increased cell proliferation.^26^ ACA11 overexpression in t(4;14)‐negative MM cells increased the number of AgNORs compared to control cells (Figure 1E, *P* < 0.001). In H929 cells, knockdown of ACA11 reduced AgNORs count in t(4;14)‐positive cells (Figure 1F, *P* = 0.02). Additionally, CD138 + cells from t(4;14)‐positive MM samples, which overexpress ACA11, had increased AgNORs counts than t(4;14)‐negative MM samples (Figure 1G, *P* = 0.03).

### 3.2 Ribosome biogenesis, cell size, and protein synthesis are upregulated by ACA11 overexpression

**Figure 2:**
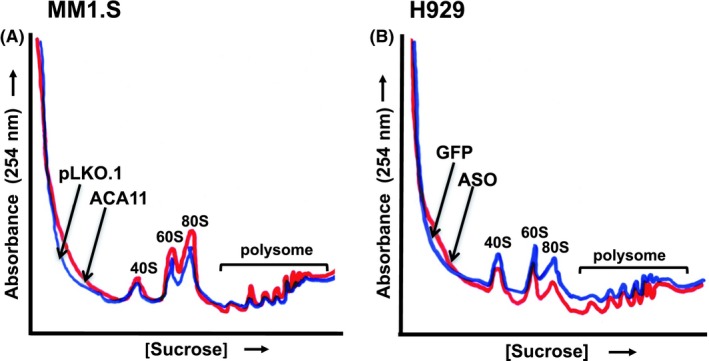
Polysome profiling analysis from multiple myeloma cell lines. Cytosolic extracts from cells were loaded onto a 7%‐47% Sucrose gradient and fractionated with recording constant UV (254 nm) monitoring. 40S, 60S, 80S and polysomes represent small and large ribosomal subunits, monosomes and multiple ribosomes per mRNA. A, MM1.S cells overexpressing ACA11 or control empty vector (pLKO.1). B, ACA11 knockdown in ACA11‐overexpressing H929 cells

**Figure 3:**
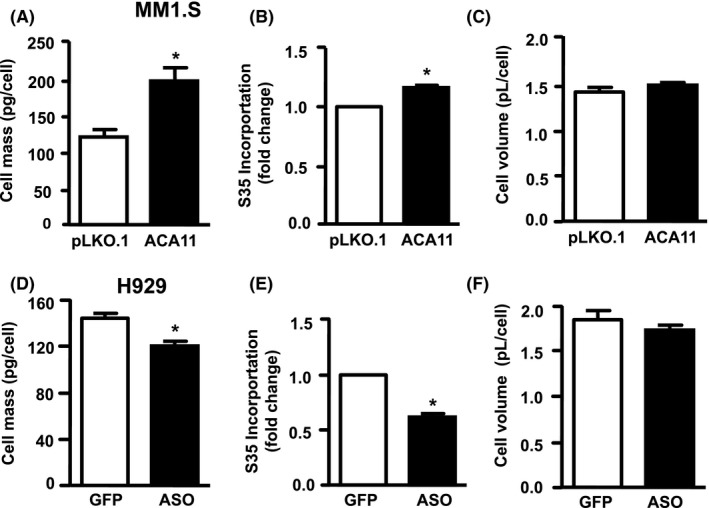
ACA11 overexpression increases cell size and protein synthesis in multiple myeloma cells. A, ACA11 overexpression in MM1.S cells is analyzed for steady‐state protein mass, (B) 35S incorporation over a 4‐h period, and (C) cell volume. D, ACA11 knockdown by antisense nucleotide (ASO) in H929 cells is analyzed for steady‐state protein mass, (E) 35S incorporation over a 4‐h period, and (F) cell volume (F)*P < 0.05

Since ACA11 regulates nucleoli characteristics in MM, and the nucleolus is the site of ribosome biogenesis in the cell, we hypothesize that ACA11 upregulates ribosome biogenesis and increases protein synthesis in MM cells. Polysome profiling of cytosolic extracts from control or ACA11 overexpressing cells demonstrated that MM cells overexpressing ACA11 had higher levels of cytosolic ribosomal subunits and actively translating polysomes compared to control infected cells (Figure 2A). Antisense knockdown of ACA11 in H929 cells reduced both individual ribosomal subunits and actively translating polysomes (Figure 2B). These findings correlated with ACA11 overexpression significantly increasing both steady‐state cell mass (Figure 3A, *P* = 0.02) and new protein synthesis, as shown by 35S incorporation (Figure 3B, *P* = 0.01). However, there was no change in steady‐state cell volume (Figure 3C, *P* = 0.26) in MM cells. These findings were further confirmed by complementary ACA11 knockdown experiments in H929 cells with decreased ACA11 expression resulting in significant reductions in steady‐state cell mass (Figure 3D, *P* = 0.007) and new protein synthesis (Figure 3E, *P* = 0.02) combined with no change in steady‐state cell volume (Figure 3F, *P* = 0.35).

### 3.3 ACA11 overexpression increases 45S pre‐rRNA, 47S pre‐rRNA levels, and protein synthesis via ROS

**Figure 4:**
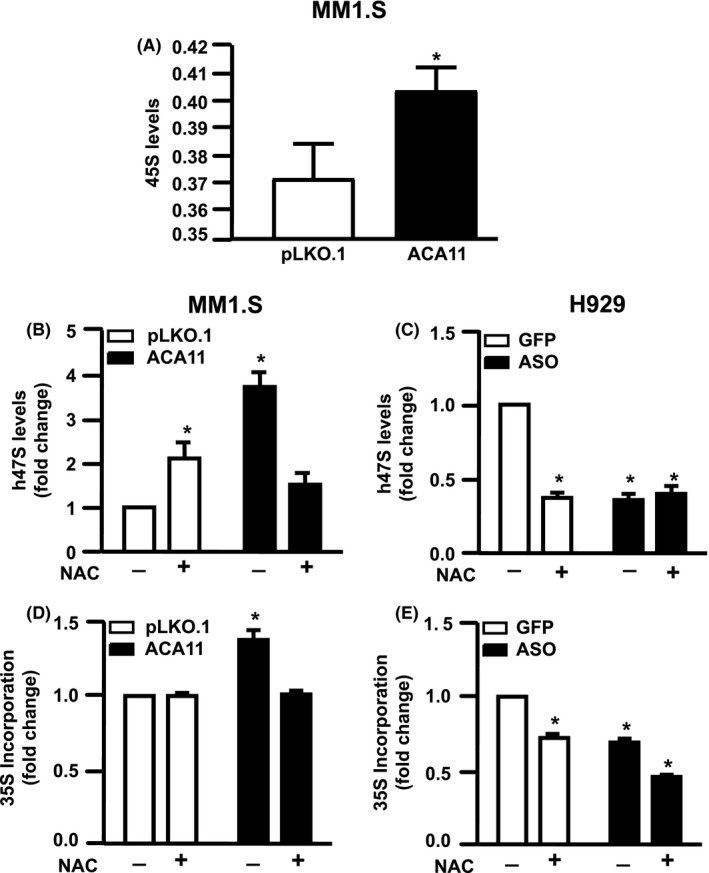
N‐acetyl‐cysteine (NAC) treatment reduces 47S levels and 35S incorporation in multiple myeloma cells lines overexpressing ACA11. A, 45S pre‐rRNA levels in MM1.S (B) 47S pre‐rRNA levels in MM1.S and (C) H929 cells analyzed by real‐time quantitative reverse transcription PCR in response to NAC (1.0 mmol/L) treatment for 24 h. D, Amount of protein synthesized at basal condition and after treatment with NAC for 24 hours in MM1.S and (E) H929 cells were measured by analyzing the amount of 35S incorporation over a 4‐h time period. *P < 0.05

The qualitative ribosomal profiling data (Figure 2) suggested that ACA11 overexpression increased ribosome biogenesis, which begins with the rate‐limiting step of the pre‐47S rRNA transcription. The mature 28S, 18S, and 5.8S rRNAs packaged with proteins in to the small and large ribosomal subunits are processed from a single 47S pre‐rRNA transcript made from the rDNA locus.^11^ We found a significant increase in the 45S pre‐rRNA levels in MM cells overexpressing ACA11 (Figure 4A, *P* = 0.02), which is considered the steady state marker of transcription.^27^, ^28^ Furthermore, given that ACA11‐ induced cell proliferation is dependent on ROS generation,^8^ we treated these cells with the antioxidant NAC. Interestingly, in MM1.S infected with empty vector (pLKO.1), treatment with NAC caused a doubling in pre‐47S rRNA levels (Figure 4B, *P* = 0.01). Scavenger of ROS with NAC attenuated pre‐47S rRNA levels to the levels of NAC‐treated control cells (Figure 4B, *P* = 0.08). Corroborating these data, ROS inhibition with NAC in MM cells which express high levels of ACA11 resulted in a significant attenuation of pre‐47S rRNA levels (Figure 4C, *P *= <0.0001). Knockdown of ACA11 attenuated pre‐47S rRNA levels to those of control cells treated with NAC (Figure 4C, *P *= <0.0001). NAC treatment in addition to ACA11 knockdown did not further reduced 47S pre‐rRNA transcript levels (Figure 4B, *P *= <0.0001).

Because ACA11 overexpression increases rRNA transcription, we addressed the hypothesis that ROS‐induced by ACA11 overexpression increases protein synthesis. ACA11 overexpression increased protein synthesis in MM cells (Figure 4D, *P* = 0.02). NAC treatment lowered 35S incorporation in ACA11 overexpressing cells to control levels (Figure 4D, *P* = 0.7), indicative of decreased protein synthesis, but did not impact control cells. We also observed that NAC treatment and ACA11 knock down decreased protein synthesis in cells (Figure 4E, *P* = 0.0004). Given the increase in cytosolic polysomes correlated with the increase in protein synthesis and 47S pre‐rRNA transcription associated with ACA11 overexpression in MM cells, we investigated the effects of ACA11 overexpression on the export of rRNA from the nucleus to the cytosol. However, we did not identify any changes on mature rRNAs being exported to the cytosol (Figure S1).

Given that post‐transcriptional changes are implicated in ribosome biogenesis we next evaluated the 2′‐O‐methylation in MM cells. The extent of methylation was analyzed for all annotated 2′‐O*‐*methylated (2′‐O‐Me) sites in human 5.8, 18, and 28S rRNA. The Meth‐Scores for these sites did not significantly change between MM1.S cells overexpressing ACA11 and empty vector (Table S2, *P* > 0.05). The reads plotted and visualized in Integrative Genomics Viewer also did not reveal significant differences in methylation status or fragmentation pattern (Figure S2), suggesting that overexpression of ACA11 does not alter the function of C/D box snoRNA machinery during ribosome biogenesis in MM cells.

### 3.4 ACA11 overexpression increases sensitivity to Btz in MM cells

**Figure 5:**
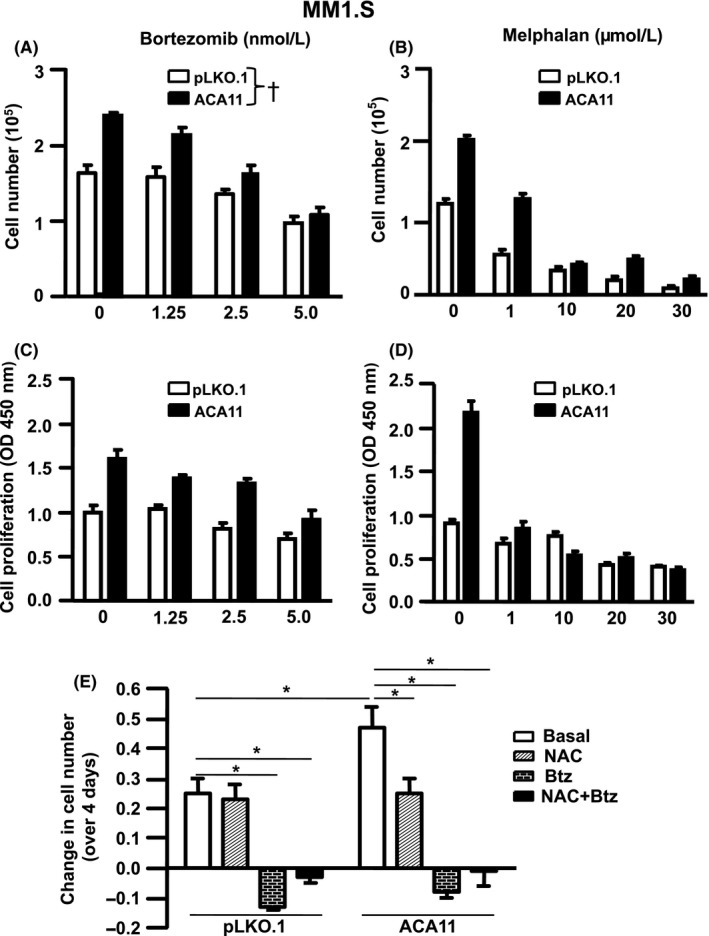
ACA11 is associated to increased response to bortezomib in MM1.S cells. A, MM1.S cells overexpressing ACA11 increased the slope curve correspondent to cell number, (B) while the slope curves were similar in the cell proliferation assay. C, Slope curves correspondent to cell number, and (D) proliferation (D) were similar between MM1.S cells infected with pLKO.1 or ACA11 after melphalan treatment. E, Change in cell number in MM1.S cells overexpressing or not ACA11 after treatment with NAC (1.0 mmol/L) and/or Bortezomib (5.0 nmol/L) over 4 d. ^†^Term for all models are given in Table 1. *P < 0.05

To determine if ACA11 overexpression is associated with responsiveness to common chemotherapy agents, we evaluated the effects ACA11 on the sensitivity to Btz and melphalan in two ways—by evaluating the dose–response relationship in terms of (a) cell growth and (b) proliferation in MM1.S cells (Figure 5A‐D). The slope of the dose–cell growth relationship was higher in ACA11 overexpressing MM1.S cells, indicating an increased sensitivity to Btz (Table 1, *P* = 0.008), and the dose of Btz necessary to kill 100% of MM cells overexpressing ACA11 (*x*‐intercept) lower (8.9 nmol/L compared to 12.3 nmol/L in pLKO.1 infected cells). However, the dose–response relationship between proliferation and Btz dose was not different between groups (Table 1, *P* = 0.06) and the doses of Btz necessary to prevent proliferation (*x*‐intercept) were similar between control cells or overexpressing ACA11 (Table 1). The slopes of the relationships between cell number and dose (Table 1, *P* = 0.33), and proliferation and dose (Table 1, *P* = 0.32) of melphalan were not different between MM overexpressing ACA11 and control, suggesting that ACA11 does not modify the response to melphalan at the doses tested here.

**Table 1:** Slope and intercept values obtained from dose–response analysis in MM1.S cells infected with pLKO.1 or ACA11 after treatment with bortezomib or melphalan

	Treatment	Condition	Slope	Intercept	*p* value
Cell number	Bortezomib	pLKO.1	−0.13 ± 0.01	12.29	0.008
		ACA11	−0.26 ± 0.01	8.29	
	Melphalan	pLKO.1	−0.02 ± 0.01	29.27	0.33
		ACA11	−0.05±0.01	29.79	
Cell proliferation	Bortezomib	pLKO.1	−0.06 ± 0.02	15.10	0.06
		ACA11	−0.13 ± 0.01	12.05	
	Melphalan	pLKO.1	−0.01 ± 0.004	55.08	0.32
		ACA11	−0.04 ± 0.023	33.94	

## 4 DISCUSSION

The orphan snoRNA ACA11 is overexpressed co‐transcriptionally with the NSD2 gene in MM patients as a result of the t(4;14) chromosomal translocation, and stimulates cell proliferation.^7^, ^8^ Here, we demonstrated for the first time that ACA11 also up regulates ribosome biogenesis in a ROS‐dependent manner. This conclusion is supported by the following lines of evidence: (a) ACA11 overexpression induced an increase in nucleoli number and size as well as in AgNOR numbers in both MM cells lines and patients; (b) ACA11 overexpression upregulated the available pool of cytosolic ribosomes in MM cell lines; (c) MM cells overexpressing ACA11 had both increased cellular mass and an increased rate of new protein synthesis; and (d) ACA11‐induced ROS drove increased protein synthesis and pre‐47S rRNA levels. In addition, we also found that ACA11 overexpression enhances the response of MM cell lines to Btz, while no significant difference was observed in the response to high doses of melphalan.

The nucleolus senses oncogenic stimuli and can activate RNA polymerase I leading to ribosomal gene transcription, cell growth and proliferation. A large nucleolar area and increased nucleolar number are observed in malignant tumor samples.^29^ We observed increased nucleolar number and area in human MM tumor samples that are t(4;14) positive and in ACA11 overexpressing cell lines. NORs are the structural and functional units of the nucleolus, where ribosomal DNA genes are clustered.^30^ Once activated, NORs are associated with a set of proteins involved in ribosome biogenesis, which activate RNA Polymerase I transcription of rDNA genes. Thus, NOR area is directly associated with both nucleolar area and rRNA transcription.^31^ The clinical relevance of NORs in MM patients has been demonstrated by correlating increased NOR number in stage III MM compared to stage I.^32^ In addition, higher NOR counts in MM patients are directly associated with poorer survival.^33^ Together these data provide evidence that ACA11 overexpression drives a key nucleolar phenotype in MM associated with t(4;14) chromosomal translocation and poor outcomes.

Given changes in the nucleoli induced by ACA11 overexpression, we next examined the effects of ACA11 levels on ribosome biogenesis by sucrose gradient polysome profiling. ACA11 overexpression in t(4;14)‐negative myeloma cells induced an increase in the 60S, 80S monosome peak, and polysome peaks suggesting an increase in mRNA translation (Figure 2A). Conversely, ACA11 knock down reduced the 40S, 60S, and 80S monosome peaks and led to a slight reduction in the polysome peaks in H929 cells (Figure 2B). The amount of ribosomes associated with polysome peaks is directly related to the global translational initiation rate.^34^ Hence, our data led us to speculate that ACA11 overexpression stimulates the global translation initiation rates in MM cells, and increased protein synthesis necessary to support increased cell growth (Figure 1B).^8^ ACA11 overexpression induced an increase in steady‐state cell mass (Figure 3A), followed by an increase in global protein synthesis, represented by 35S incorporation in these cells (Figure 3B). Consistently, ACA11 knock down was followed by a decrease in both cell mass (Figure 3D) and nascent protein synthesis per cell (Figure 3E). Our findings are consistent with the expanding appreciation of the role of snoRNAs in normal and malignant hematology. For example, C/D box snoRNAs and RNA 2′‐O‐methylation are critical for AML‐ETO induced leukemia stem cell activity.^14^ The tumor suppressor p53 also has been found to regulate the RNA methylase fibrillarin.^13^

The precursor 45S is considered the unique steady state marker of transcription because the primary transcript pre‐47S is rapidly processed.^27^, ^28^ Here we observed higher levels of 45S pre‐rRNA in MM cells overexpressing ACA11 (Figure 4A) compared to controls. Consistently, in other malignancies such as cervical and prostate cancer, increased 45S levels were also described.^35^, ^36^ Previously, we found that the pro‐proliferative effects of ACA11 overexpression are ROS‐dependent 8 and the current study demonstrates the role of ROS in ribosome biosynthesis in MM cells. ACA11 overexpression increased pre‐47S rRNA levels. Treatment with NAC induced a significant increase in 47S levels in MM1.S cells infected with empty vector but reduced the 47S levels in ACA overexpressing cells (Figure 4A). In agreement with these observations, in H929 cells we found that both NAC treatment and ACA11 knock down reduced the pre‐47S rRNA levels (Figure 4B). In parallel, we also evaluated if the increase in protein synthesis induced by ACA11 overexpression was induced by ROS generation. In fact, in MM1.S cells infected with pLKO.1, we found that NAC treatment induced a small, but significant increase in protein synthesis, but the higher protein levels induced by ACA11 overexpression were normalized after NAC treatment (Figure 4C). In addition, in H929 cells which have high levels of ACA11 in both NAC treatment and ACA11 knock down reduced protein synthesis (Figure 4D). High levels of pre‐47S rRNA transcripts have been reported in cancer cell lines and patients.^9^, ^37^, ^38^ The result of increased pre‐47S levels after NAC treatment in MM1.S transduced with empty vector was unexpected, but paradoxal effects of antioxidant treatments in other malignancies has been reported rRNA transcription.

Myeloma patients with t(4;14) have poor overall survival, but they are notably sensitive to proteasome inhibitors. Our data demonstrate that ACA11 overexpression contributes to the chemosensitivity of MM cells to proteasome inhibitors. Given the increase in protein synthesis by driven high ACA11 levels, we hypothesized that ACA11 overexpressing cells would be especially sensitive the proteasome inhibitor Btz. ACA11 overexpression increased the response to Btz in MM cells, as evidenced by both the higher slope of the relationship between cell number and Btz dose (Table 1), and the lower Btz dose (8.9 nmol/L, Table 1) required to kill 100% of cells overexpressing ACA11. There was also a trend toward decreased cell proliferation in cells overexpressing ACA11 (*P* = 0.06, Table 1). However, we detected no significant difference in the response to the alkylating agent melphalan in cells overexpressing ACA11 (Table 1). Our data suggest that increased ribosome biogenesis and protein synthesis put extra demand on proteasomes, making cells especially sensitive to their inhibition, and perhaps leading to toxic levels of ROS and cell death. While we did not find that ACA11 induce resistance to the alkylating agent melphalan, the doses we used here were those cited for use in cell culture.^23^ Doses of drugs used in cell culture experiments are sustained unlike the levels achieved in patients.^39^, ^40^, ^41^ The doses of Btz (1.25‐5.0 nmol/L) used in these experiments were two orders of magnitude lower than peak plasma levels seen in patients (421‐692 nmol/L).^40^, ^41^ In contrast, the doses we used for melphalan were approximately the same as the peak plasma dose achieved (4.8‐42 µmol/L) in patients.^39^ Therefore, important differences in response to melphalan may be seen with lower doses. Our results explain in part the clinical behavior of t(4;14) MM which, while associated with poor outcome, tends to be remarkably sensitive to proteasome inhibition. These data suggest that treatments targeting oxidative stress could be used cooperating with proteasome inhibitors to individualize the therapy for MM patients with the t(4;14) chromosomal translocation. Specifically, chemotherapy agents that scavenge or increase ROS could be used in patients with t(4;14) at different stages, during induction of treatment with proteasome inhibitor, or during maintenance therapy, respectively. Future work will elucidate the potential for ACA11 to cooperate with the protein coding NSD2 histone methyltransferase activated by the t(4;14).

## CONFLICT OF INTEREST

The authors declare no competing financial interests.

## AUTHOR CONTRIBUTIONS

V.O., N.M., C.T., K.Q.K., H.S.Z., and L.B.M. performed the experiments; V.O., N.M., M.L.B., C.T., K.Q.K., H.S.Z., L.B.M., and M.H.T. analyzed the results. V.O., N.M., K.Q.K., H.S.Z. M.L.B., and L.B.M. made the figures. N.M., M.L.B., H.S.Z., L.B.M., and M.H.T. designed the research. V.O., N.M., M.L.B., L.B.M., and M.H.T. wrote the paper.
