# Supplementary figures and images for: The snoRNA target of t(4;14) in multiple myeloma regulates ribosome biogenesis
Source: FASEB Bioadv. 2019 May 22;1(7):404–14. doi: 10.1096/fba.2018-00075 (PMC6996358; doi:10.1096/fba.2018-00075)

# MM1.S

**A**

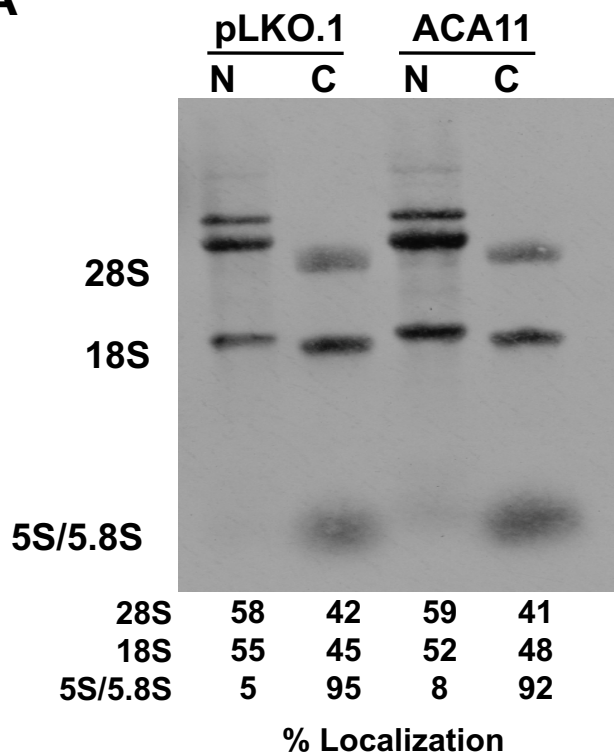

a-Lamin A/C

a-SOD

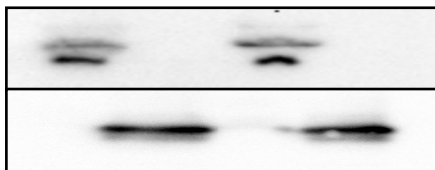

# H929

**B**

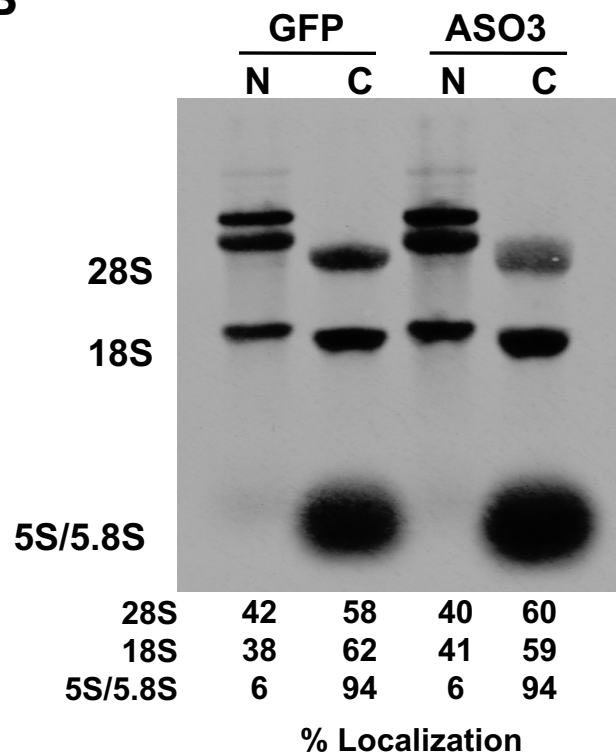

a-Lamin A/C

a-SOD

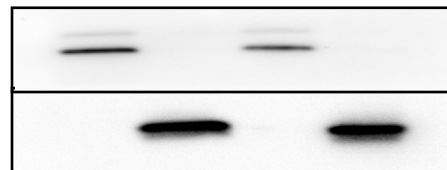

Supplement: Supplementary file 1 [file FBA2-1-404-s001.pdf]

# MM1.S cells

pLKO.1

ACA11

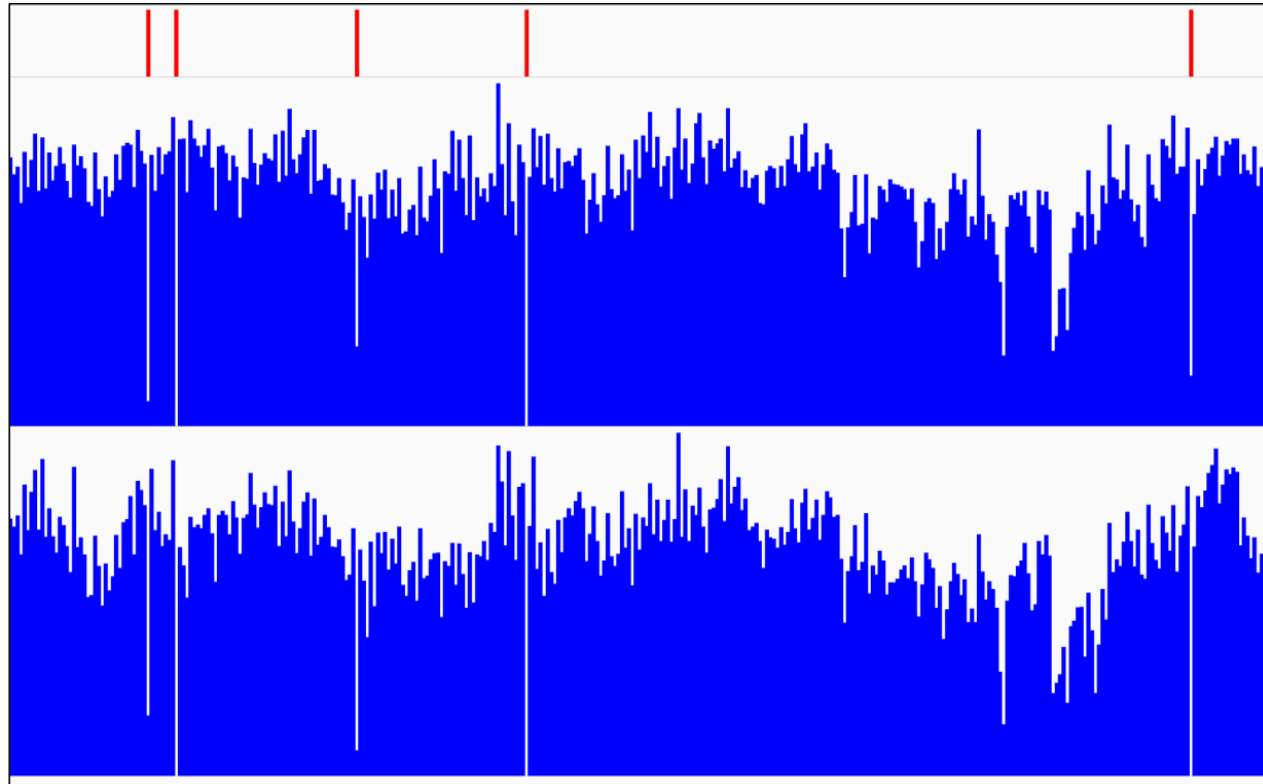

Supplement: Supplementary file 2 [file FBA2-1-404-s002.pdf]
