# Supplementary material for: The snoRNA target of t(4;14) in multiple myeloma regulates ribosome biogenesis
Source: FASEB Bioadv. 2019 May 22;1(7):404–14. doi: 10.1096/fba.2018-00075 (PMC6996358; doi:10.1096/fba.2018-00075)
Supplement: Supplementary file 3 [file FBA2-1-404-s003.pdf]

| | |
|---|---|
| | **5'rAppCTGTAGGCACCATCAAT/3ddC/ 3'** |
| **RS-1 (RT Primer)** | /5Phos/AGATCGGAAGAGCGTCGTGTAGGGAAAGA<u>GTGTAGATCTCGGTGGTCGC</u>/iSp18/ CACTCA /iSp18/*TTCAGACGTGTGCTCTTCCG*ATCT**ATTGATGGTGCCTACAG** |
| **RS-F (FW primer)** | AATGATACG<u>GCGACCACCGAGATCTACAC</u> |
| **RS-R-1** | CAAGCAGAAGACGGCATACGAGAT***<u>AGTCGT</u>***GTGACTGGAG*TTCAGACGTGTGCTCTTCCG* |
| **RS-R-2** | CAAGCAGAAGACGGCATACGAGAT***<u>ACTGAT</u>***GTGACTGGAG*TTCAGACGTGTGCTCTTCCG* |
| **RS-R-3** | CAAGCAGAAGACGGCATACGAGAT***<u>ATGCTG</u>***GTGACTGGAG*TTCAGACGTGTGCTCTTCCG* |
| **RS-R-4** | CAAGCAGAAGACGGCATACGAGAT***<u>ACGTCG</u>***GTGACTGGAG*TTCAGACGTGTGCTCTTCCG* |
| **RS-R-5** | CAAGCAGAAGACGGCATACGAGAT***<u>AGCTGC</u>***GTGACTGGAG*TTCAGACGTGTGCTCTTCCG* |
| **RS-R-6** | CAAGCAGAAGACGGCATACGAGAT***<u>ATCGTA</u>***GTGACTGGAG*TTCAGACGTGTGCTCTTCCG* |
| **RS-R-7** | CAAGCAGAAGACGGCATACGAGAT***<u>TGGTCA</u>***GTGACTGGAG*TTCAGACGTGTGCTCTTCCG* |
| **RS-R-8** | CAAGCAGAAGACGGCATACGAGAT***<u>CACTGT</u>***GTGACTGGAG*TTCAGACGTGTGCTCTTCCG* |
| **RS-R-9** | CAAGCAGAAGACGGCATACGAGAT***<u>ATTGGC</u>***GTGACTGGAG*TTCAGACGTGTGCTCTTCCG* |
| **RS-R-10** | CAAGCAGAAGACGGCATACGAGAT***<u>GATCTG</u>***GTGACTGGAG*TTCAGACGTGTGCTCTTCCG* |
| **RS-R-11** | CAAGCAGAAGACGGCATACGAGAT***<u>TCAAGT</u>***GTGACTGGAG*TTCAGACGTGTGCTCTTCCG* |
| **RS-R-12** | CAAGCAGAAGACGGCATACGAGAT***<u>CTGATC</u>***GTGACTGGAG*TTCAGACGTGTGCTCTTCCG* |

**Supplemental table 1.** Linker and Primers used for 2'-O-Methylation-seq library preparation
