# Supplementary material for: The snoRNA target of t(4;14) in multiple myeloma regulates ribosome biogenesis
Source: FASEB Bioadv. 2019 May 22;1(7):404–14. doi: 10.1096/fba.2018-00075 (PMC6996358; doi:10.1096/fba.2018-00075)
Supplement: Supplementary file 4 [file FBA2-1-404-s004.pdf]

rRNA	Control		ACA11 overexpressing	
5.8S				
**14**	0.340	0.069	0.168	0.290
**157**	0.996	0.004	0.999	0.001
18S				
**27**	1.000	0.000	1.000	0.000
**99**	0.983	0.003	0.983	0.002
**116**	0.977	0.007	0.970	0.019
**121**	0.965	0.021	0.959	0.027
**159**	0.995	0.001	0.994	0.006
**166**	0.999	0.000	0.995	0.009
**172**	0.995	0.002	0.989	0.007
**174**	0.893	0.029	0.916	0.053
**428**	0.794	0.037	0.727	0.111
**436**	0.826	0.048	0.755	0.127
**462**	0.995	0.003	0.998	0.003
**468**	0.983	0.003	0.985	0.004
**484**	0.997	0.004	0.996	0.007
**509**	1.000	0.001	1.000	0.000
**512**	0.998	0.001	1.000	0.000
**517**	0.998	0.002	0.998	0.002
**576**	0.984	0.006	0.962	0.038
**590**	0.993	0.003	0.995	0.004
**601**	1.000	0.000	0.996	0.004
**627**	0.999	0.000	0.998	0.000
**644**	0.997	0.002	0.997	0.002
**668**	0.993	0.005	0.996	0.001
**683**	0.999	0.001	0.999	0.001
**797**	0.985	0.002	0.992	0.003
**799**	1.000	0.000	1.000	0.000
**867**	0.612	0.083	0.630	0.099
**1031**	1.000	0.000	1.000	0.000
**1272**	0.861	0.055	0.877	0.040
**1288**	0.998	0.000	0.998	0.001
**1328**	1.000	0.000	1.000	0.000
**1383**	0.998	0.001	0.998	0.002
**1391**	0.999	0.000	0.999	0.001
**1442**	0.991	0.008	0.994	0.004
**1490**	1.000	0.000	1.000	0.000
**1678**	0.998	0.001	0.999	0.001
**1703**	0.994	0.002	0.991	0.008
**1804**	0.968	0.008	0.963	0.024
28S				
**1340**	0.910	0.022	0.923	0.015
**1522**	1.000	0.000	1.000	0.000
**1524**	1.000	0.000	1.000	0.000

**1534**	1.000	0.000	0.997	0.002
**1625**	0.999	0.001	0.998	0.001
**1871**	0.992	0.007	0.996	0.006
**2351**	0.974	0.027	0.997	0.002
**2363**	1.000	0.000	1.000	0.000
**2364**	1.000	0.000	0.997	0.006
**2365**	0.794	0.129	0.922	0.027
**2415**	0.933	0.023	0.944	0.009
**2422**	0.991	0.003	0.988	0.009
**2424**	0.998	0.002	1.000	0.000
**2787**	0.981	0.006	0.983	0.003
**2804**	0.986	0.005	0.986	0.010
**2815**	0.991	0.003	0.995	0.004
**2824**	0.988	0.004	0.991	0.006
**2837**	1.000	0.000	1.000	0.000
**2861**	0.901	0.031	0.879	0.088
**3701**	0.999	0.001	1.000	0.000
**3718**	0.998	0.001	1.000	0.001
**3724**	0.981	0.024	0.996	0.001
**3744**	0.975	0.007	0.990	0.005
**3760**	1.000	0.001	1.000	0.000
**3785**	1.000	0.000	1.000	0.000
**3808**	0.979	0.004	0.993	0.008
**3818**	1.000	0.000	0.999	0.001
**3825**	0.999	0.000	0.998	0.002
**3830**	0.997	0.002	0.993	0.003
**3841**	0.992	0.002	0.993	0.003
**3869**	0.969	0.007	0.972	0.005
**3887**	0.987	0.018	0.996	0.003
**3925**	0.983	0.004	0.976	0.028
**3944**	0.991	0.005	0.996	0.004
**4042**	0.416	0.123	0.726	0.118
**4054**	0.995	0.004	1.000	0.000
**4196**	1.000	0.001	1.000	0.000
**4227**	0.996	0.001	1.000	0.000
**4228**	1.000	0.000	1.000	0.000
**4306**	0.997	0.006	1.000	0.000
**4370**	0.999	0.001	0.999	0.001
**4392**	0.999	0.000	0.999	0.001
**4456**	0.990	0.005	0.978	0.015
**4494**	0.999	0.001	1.000	0.001
**4498**	0.867	0.010	0.904	0.057
**4499**	0.978	0.002	0.988	0.003
**4523**	0.999	0.000	0.999	0.001
**4536**	0.995	0.005	0.997	0.004
**4571**	0.986	0.002	0.979	0.007

**4590**	0.851	0.022	0.832	0.095
**4618**	0.940	0.005	0.939	0.034
**4620**	0.934	0.018	0.915	0.028
**4623**	0.998	0.001	0.994	0.006
**4637**	0.946	0.006	0.940	0.019

Supplemental table 2. Meth-Scores for annotated 2'-O-Me sites in 5.8, 18, and 28S rRNA from control and ACA11 overexpressing MM.1S cells. Scores are shown as the mean score|standard deviation.
