# Supplementary material for: The snoRNA target of t(4;14) in multiple myeloma regulates ribosome biogenesis
Source: FASEB Bioadv. 2019 May 22;1(7):404–14. doi: 10.1096/fba.2018-00075 (PMC6996358; doi:10.1096/fba.2018-00075)
Supplement: Supplementary file 5 [file FBA2-1-404-s005.docx]

**Figure S1.** Autoradiography of rRNA export in MM cell lines overexpressing ACA11. (A) MM1.S cells or (B) H929 cells were labeled with [methyl3 H]methionine for 30 minutes and chased for 2 hours. 2x10^6^ cells were subjected to fractionation into cytoplasmatic (C) and nucleolar (N) extracts. Total RNA extracted from each condition was transferred onto membranes and submitted to autoradiography

**Figure S2.** 2’-O-Methylated (2’-O-Me) sites evaluated in MM1.S cells overexpressing ACA11. 2’-O-Me sites can be seen when plotted. Reads from control and ACA11 overexpressing MM.1S cells were plotted in log scale in IGV with annotated 2’-O-Me sites marked by red bars. This representative snapshot encompasses positions 1200-1500 of the 18S rRNA
